# Depth and Seasonality of Soil Respiration in *Caragana korshinskii* Plantation on the Loess Plateau

**DOI:** 10.3390/plants14193038

**Published:** 2025-10-01

**Authors:** Yarong Sun, Yunming Chen

**Affiliations:** 1 Institute of Soil and Water Conservation, College of Soil and Water Conservation Science and Engineering, Northwest A&F University, Yangling, Xianyang 712100, China; twoslcyouth@163.com; 2 Institute of Soil and Water Conservation, Chinese Academy of Sciences and Ministry of Water Resources, Yangling, Xianyang 712100, China

**Keywords:** carbon dioxide, soil depth, seasons, greenhouse gas, climate change

## Abstract

Quantifying deep soil (10–100 cm) and non-growing-season soil respiration (SR) is crucial for refining carbon (C) cycle models, yet the regulatory mechanisms governing these processes remain unclear. The novelty of this study lies in its focus on deep soils and non-growing seasons to elucidate how soil properties regulate SR under these special conditions. We conducted an on-site field experiment in the *Caragana korshinskii* plantation, measuring SR at soil depths of 0–10 cm, 10–50 cm, and 50–100 cm during the non-growing season and growing. The results suggested that the annual cumulative soil CO_2_ fluxes reached 510.1 (0–10 cm), 131.5 (10–50 cm), and 45.3 g CO_2_·m^−2^ (50–100 cm). These emissions during the non-growing season accounted for 33%, 31%, and 32%, respectively. The soil physical properties (temperature, moisture, bulk density) explained the greatest variation in SR during growing and non-growing periods, followed by the biological properties (α-diversity, root biomass) and chemical properties (soil organic C, ammonium nitrogen, total C/nitrogen ratio). Depth-specific analysis demonstrated that soil physical properties explained the most SR variance at three depths with independent contributions of 78.9% (0–10 cm), 89.7% (10–50 cm), and 76.9% (50–100 cm). These values exceeded the independent contributions of chemical properties (70.3%, 70.9%, 60.0%) and biological properties (54.9%, 45.1%, 41.6%) at the corresponding depths. Overall, deep soil and non-growing season SR represent important C emission sources; excluding them may therefore substantially overestimate net C sequestration potential.

## 1. Introduction

Soil respiration (SR) constitutes a central role in the global carbon (C) cycle, serving as the primary pathway through which photosynthetically fixed CO_2_ is emitted into the atmosphere [1]. Globally, SR contributes approximately 98 ± 12 Pg C annually [2], and even minor fluctuations in SR can substantially alter atmospheric CO_2_ concentrations, with important implications for climate–C feedback [3]. This underscores the need to improve our understanding of SR dynamics to accurately predict carbon-climate feedbacks under changing environmental conditions. Notably, although most studies have focused on SR during the growing season, it has traditionally been assumed that non-growing season SR is negligible. However, recent studies [4,5] suggest that microbial activity and soil respiration persist throughout the year, decomposing organic matter and releasing CO_2_ even during dormancy. This challenges the traditional view and highlights the potential underestimation of C fluxes when non-growing season SR is overlooked, which could lead to distorted predictions of C-climate interactions.

While SR from surface soils (0–10 cm) has been extensively investigated, SR from deeper soil layers (10–100 cm) remains poorly understood [6,7,8]. The theoretical framework for understanding SR dynamics in deep soils is limited, as previous models often assume two main points: (1) deep SR is negligible, and (2) carbon in deep soils is inert [9]. However, recent findings challenge these assumptions. For instance, Sun et al. (2025) [10] reported that subsurface layers (10–100 cm) contributed nearly one-third of total soil CO_2_ efflux in a Robinia pseudoacacia plantation. Similarly, warming experiments have revealed enhanced C mineralization in deep soils, indicating their potential vulnerability to climate change [11,12]. This suggests that a deeper understanding of deep soil SR dynamics is essential for accurately predicting the response of carbon cycles to climate change.

SR in surface soils is regulated by soil physicochemical conditions, plant traits, and microbial communities [13,14,15,16]. Notably, the mechanisms regulating SR likely differ substantially between surface and deep soils: deep soils are characterized by lower nutrient availability, sparser root biomass, and distinct microbial composition [17,18], which may shift the dominant regulators of SR. Similarly, seasonal changes further reshape SR regulating mechanisms: shifts in temperature and precipitation across seasons alter plant activity, substrate inputs to soils, and microbial metabolic processes [19]. These seasonal dynamics may not only change the strength of individual drivers but also rearrange their relative importance for SR—yet how such seasonal shifts interact with depth-related differences in regulating mechanisms remains unaddressed. By addressing this knowledge gap, our study aims to provide insights that will enhance the understanding of C-climate feedbacks and inform better management of soil C storage.

The Loess Plateau of China is the world’s largest loess deposition region, marked by severe soil erosion, rugged topography, and deep loess layers [20]. Notably, the Zhifanggou watershed is widely recognized as a representative catchment of the Loess Hilly Region, which typifies the broader Loess Plateau. It encompasses the characteristic geomorphology, soil types, and ecological evolution processes of this region [21,22,23]. *Caragana korshinskii* Kom. (Fabaceae) is widely planted due to its robust root system and high biomass yield. At present, *C. korshinskii* plantations cover more than 5500 ha within this region [24], providing a representative case for understanding ecosystem processes and the ecological effects of vegetation restoration across the Loess Plateau [25].

Therefore, continuous measurements of soil CO_2_ concentrations using CO_2_ probes were performed at 0–10 cm, 10–50 cm, and 50–100 cm depths within a C. korshinskii plantation in the Zhifanggou watershed from January to December 2023. This study addresses the critical knowledge gap in SR by integrating seasonal and depth-resolved measurements, providing a unique opportunity to explore the drivers of SR across different soil depths and during the non-growing season. Our research aimed to address two questions: (1) How do SR dynamics differ between two seasons, and what is the relative contribution of each depth to annual C release? (2) How do plant–soil properties influence SR across depths and seasons? We hypothesize that (1) SR will decrease with increasing soil depth due to lower nutrient availability, microbial activity, and root biomass. (2) During the growing season, SR is expected to increase due to higher soil temperature (ST), increased plant activity, and enhanced microbial metabolism.

The innovation of this study lies in its integration of seasonal and depth-resolved SR measurements, offering new insights into the dynamics of SR across different soil depths and seasonal gradients. This study helps fill a critical gap in our understanding of soil carbon cycling, particularly in deep soils and during the non-growing season. By addressing these knowledge gaps, the findings will improve predictions of carbon fluxes and enhance the understanding of C-climate feedbacks in the Loess Plateau and similar semi-arid regions, which are highly sensitive to climate change.

## 2. Materials and Methods

### 2.1. Site Description

The research is situated in Ansai County, Shaanxi Province, China (108°5′44″ E–109°26′18″ E and 36°30′45″ N–37°19′3″ N). The soil is classified as Calcic Cambisols [26] (FAO, 2020). The primary growing season extends from April through October, while plantations are dormant from November through March. Annual precipitation averages 528.8 mm, while the mean air temperature is 8.8 °C [27]. Elevation ranges between 1012 m and 1072 m, and climate is typical of a temperate continental zone.

### 2.2. Measurements of Soil Moisture, Soil CO_2_ Concentration, and Soil Temperature

We excavated three replicated vertical trenches (1 m × 1 m × 1 m) with a horizontal spacing of less than 8 m within the 0–100 cm soil depths (20 m × 20 m). CO_2_ probes (GMP343; Vaisala, Finland; length: 5.5 cm, diameter: 18 cm) were horizontally installed at soil depths of 10 cm, 50 cm, and 100 cm to measure CO_2_ concentration. An additional probe was deployed in the trench headspace to monitor atmospheric CO_2_ concentration. Each probe was encapsulated in a breathable polyethylene tube with sealed ends to prevent water ingress while allowing gas exchange with the surrounding soil [28]. Before deployment, the probes were calibrated using zero calibration, exposing them to a CO_2_-free environment to ensure they read zero in the absence of CO_2_. Adjacent to the CO_2_ probes, combined ST and SM sensors (CS655; Campbell Scientific, Logan, UT, USA) were installed to measure the SM and ST. The CS655 utilizes time-domain reflectometry (TDR) technology, ensuring accurate SM measurements even in partially frozen soils [29]. All sensors recorded hourly measurements from January to December 2023, including ST (°C), SM (%), and CO_2_ concentration (ppm). Notably, the instruments maintained reliable operation even after exposure to extreme weather events, though routine maintenance was performed approximately every two months to ensure accuracy and prevent drift or malfunction.

### 2.3. Soil Sampling and Analysis

Soil sampling was taken during the growing season (June 2023) and the dormant season (December 2023). Soil specimens were obtained employing a gravity coring device at three depth intervals. Subsequently, the collected soil samples were subjected to dry sieving through a 2 mm sieve. Finally, all samples were promptly transported to the lab using an ice-packed incubator to preserve their freshness. Subsequently, each specimen was split into two parts. One part was maintained at –20 °C to measure microbial biomass carbon (MBC), ammonium nitrogen (NH_4^+^_), microbial community composition and diversity, and nitrate nitrogen (NO_3^−^_). The other part was air-dried in preparation for analytical measurements including total nitrogen (TN), pH, total phosphorus (TP), SOC. Additionally, root samples were extracted at three soil depths using a root auger. Fine roots were collected manually. All residues, including stem materials, litter fragments, and dead roots, were carefully removed with tweezers. Following collection, the root specimens were carefully washed with deionized water and then oven-dried at 60 °C for 48 h to measure their biomass.

Soil properties were measured following the standardized procedures outlined by Carter and Gregorich (2007) [30]. TP content was measured colorimetrically using the ascorbic acid-molybdate method. Soil bulk density (SD) was measured using the ring knife method. SOC content was measured using the dichromate oxidation method. The extraction of soil NH_4^+^_ and NO_3^−^_ was accomplished with 2 M KCl, followed by quantification through the utilization of an Autoanalyser-3. TN content was measured using the Kjeldahl method. pH value of soil was measured using a pH meter with a 1:2.5 soil-to-water ratio. Chloroform fumigation extraction was used to measure MBC content. Microbial community composition and diversity were assessed using DNA extraction followed by high-throughput sequencing [31]. Further methodological details are provided in the Supplementary Materials.

### 2.4. Calculations

Fick’s first law [32] in one dimension was used to calculate SR:

$$\mathrm{SR}\;=\;-D_{s}\frac{\Delta C}{\Delta Z}$$

D_s_ = ε D_a_

ε = (g − SM)^2.5^φ^−1^

(1) $$g=(1-\frac{SD}{m})\times 100\%$$

where D_a_, the CO_2_ diffusion coefficient at standard pressure, D_a_ = 1.47 × 10^−5^ m^2^·s^−1^. Key measured terms comprised: ΔZ, the vertical distance between those two sensors (m); D_s_, soil CO_2_ diffusivity (m^2^·s^−1^); ΔC, the difference in CO_2_ concentration between two adjacent sensors (μmol·m^−3^). For the surface flux, we calculated the gradient between the atmosphere (0 cm) and the shallowest soil sensor (10 cm). SM, soil moisture content (cm^3^·cm^−3^); m, particle density, m = 2.65 g·cm^−3^ [33]; g, gas diffusivity; SD, soil bulk density (g·cm^−3^) (Table 1); and φ, soil porosity.

The total soil CO_2_ emission was determined through integration of CO_2_ flux measurements, with seasonal cumulative values calculated for both periods using the following method:

(2) $$S=44\times 10^{-6}\times 3600\times \sum_{i}^{n}SR_{i}$$

where the 10^−6^ and 3600, the conversion coefficients; 44, the CO_2_ molar mass (g·mol^−1^); SR_i_, the soil respiration during the measurement period ti; S, the total accumulated CO_2_ emissions from soil.

An exponential model was used for the ST–SR relationship [10,34], a polynomial function was used for the SM–SR relationship [35,36], and a compound function was used for the combined ST and SM–SR relationship [37].

(3) $$SR=ae^{bST}$$

(4) $$SR=d{SM}^{2}+eSM+c$$

(5) $$\mathrm{SR}=ae^{bST}\times \left(d{SM}^{2}+eSM+c\right)$$

where a, c, d, e are constant coefficients. b, represents the temperature sensitivity.

### 2.5. Statistical Analysis

Prior to correlation and hierarchical partitioning analyses, all predictor variables were standardized (z-score transformation) to eliminate the influence of differing measurement scales. A two-way ANOVA was employed to determine the influence of seasons (growing and non-growing) and depth (0–10, 10–50, and 50–100 cm) on SR and soil chemical properties (TP, TN, NH_4^+^_, C:P, NO_3^−^_, pH, N:P, SOC, and C:N), soil physical properties (SM, ST, and SD), and soil biological properties (RB, MBC, fungal and bacterial Shannon index, fungal and bacterial community composition) using SPSS23.0. Multiple comparisons were performed with LSD tests (*p* < 0.05). Pearson correlation was utilized to analyze the correlations between SR and soil properties to select the important factors using Origin 2025. Collinearity diagnosis was used to exclude some factors with a variance inflation factor > 10 using SPSS23.0. The retained predictors included ST, SM, SD, SOC, C:P, NH_4^+^_, RB, fungal and bacterial Shannon index. To determine individual variable contributions to SR, we performed hierarchical partitioning analysis using the R package 4.1.1 ‘rdacca.hp’ [38]. Additionally, the first component of nonmetric multidimensional scaling (NMDS) depicted the fungal and bacterial community composition; the Shannon index illustrated the fungal and bacterial α-diversity.

## 3. Results

### 3.1. Variations in Soil Respiration

**Figure 1:**
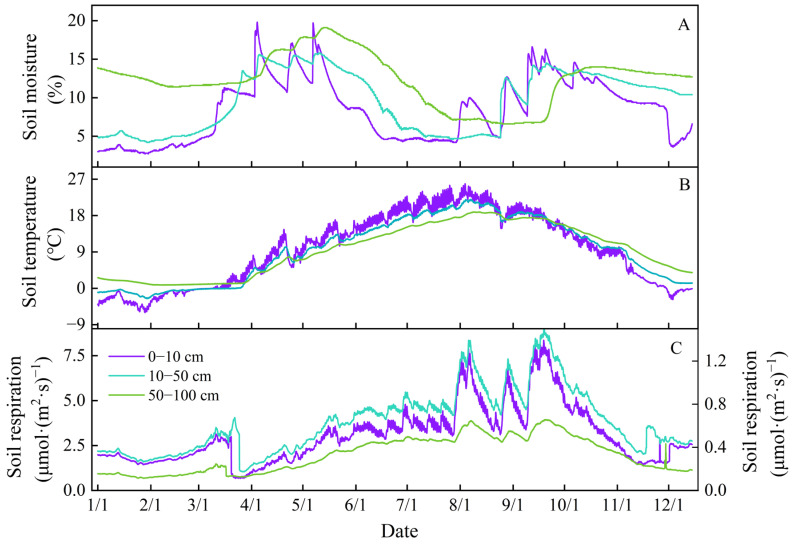
Changes in soil moisture (**A**), soil temperature (**B**), and soil respiration (**C**) in the 0–10, 10–50, and 50–100 cm depths from January to December 2023.

SR exhibited a similar multi-peak variation at 0–10, 10–50, and 50–100 cm depths. The annual mean SR values at these depths were 3.0 (0–10 cm), 0.6 (10–50 cm), and 0.3 μmol·m^−2^·s^−1^ (50–100 cm), respectively. The mean SR values were 3.3 (0–10 cm), 0.7 (10–50 cm), and 0.3 μmol·m^−2^·s^−1^ (50–100 cm) during the growing season, which were 1.4 to 3.0 times as high as those values in non-growing (Figure 1). Notably, SR reduced significantly with enhancing depth (*p* < 0.05) (Figure 1C).

**Figure 2:**
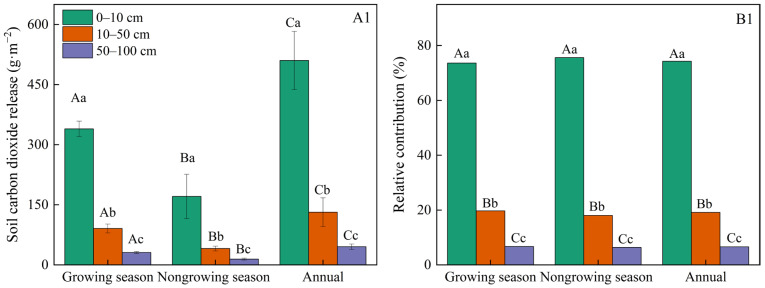
Cumulative of CO_2_ release (**A1**) and relative contribution (**B1**) during the annual, non-growing, and growing seasons at three soil depths. Capital letters above the mean values denote statistical differences between seasons (*p* < 0.05). Lowercase letters above the mean values denote statistical differences among depths (*p* < 0.05).

Cumulative CO_2_ efflux over one year reached 686.9 g CO_2_·m^−2^. Specifically, the CO_2_ effluxes for the three soil depth layers were 510.1 (0–10 cm), 131.5 (10–50 cm), and 45.3 g CO_2_·m^−2^ (50–100 cm), respectively. During the non-growing season, CO_2_ emissions represented 32.9% of the yearly total (Figure 2). Those findings suggested that CO_2_ release was underestimated if the deep soil and non-growing season SR were disregarded.

### 3.2. Soil Temperature and Moisture Regulating Soil Respiration

ST explained 2–77% of the variability in SR. Notably, the relationship between ST and SR was poorly characterized at 0–10 cm soil depth during growing season (*p* > 0.05). In contrast, frozen soil conditions significantly enhanced ST-SR coupling in the 10–50 cm depths, explaining 66% of SR variation (Table S1).

SM accounted for a proportion of the variation (2–56%) in SR at three soil depths and its relationship increased with increasing soil depth (Table S2). Notably, the R^2^ value between SM and SR was markedly greater during non-growing seasons compared to growing seasons, indicating stronger environmental coupling in colder months.

Additionally, ST and SM explained 11–90% of the variability in SR. Notably, the combined effect of ST and SM on SR was greater than that of each individual factor (Table S3)

### 3.3. Environmental Influences on Soil Respiration During Two Seasons

**Figure 3:**
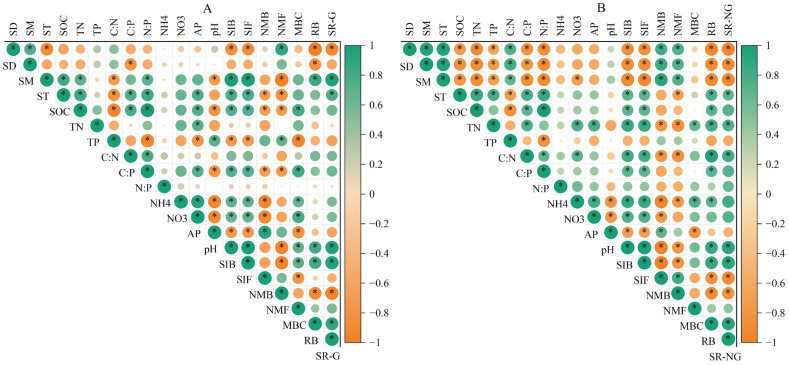
Pearson correlations between soil respiration and soil property in growing (**A**) and non-growing (**B**) seasons. The color of each circle is proportional to the value of Pearson’s correlation coefficient. Orange indicates a negative relationship; Green indicates an active relationship. “*” indicated a statistical difference (*p* < 0.05). TN, total nitrogen; SOC, soil organic carbon; TP, soil total phosphorus; N:P, the ratio of total nitrogen/total phosphorus; C:N, the ratio of soil organic carbon/total nitrogen; C:P, the ratio of soil organic carbon/total phosphorus; NH_4^+^_, ammonium nitrogen; NO_3^−^_, nitrate nitrogen; pH, potential of hydrogen; SD, soil bulk density; ST, soil temperature; SM, soil moisture; RB, root biomass; MBC, microbial biomass carbon; SIB, bacterial Shannon index; SIF, fungal Shannon index; NMDS1B, bacterial the first component of nonmetric multidimensional scaling 1 analysis; NMDS1F, fungal the first component of nonmetric multidimensional scaling 1 analysis; SR-G, soil respiration during the growing season; SR-NG, soil respiration during the non-growing season. * *p* < 0.05.

**Figure 4:**
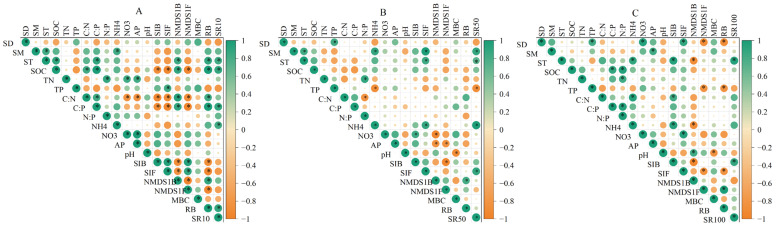
The relative importance of predictive factors in regulating soil respiration at depths of 0–10 (**A**), 10–50 (**B**), and 50–100 cm (**C**). “*” indicated a significant difference (*p* < 0.05). TN, total nitrogen; SOC, soil organic carbon; TP, soil total phosphorus; N:P, the ratio of total nitrogen/total phosphorus; C:N, the ratio of soil organic carbon/total nitrogen; C:P, the ratio of soil organic carbon/total phosphorus; NH_4^+^_, ammonium nitrogen; NO_3^−^_, nitrate nitrogen; pH, potential of hydrogen; SD, soil bulk density; ST, soil temperature; SM, soil moisture; RB, root biomass; MBC, microbial biomass carbon; SIB, bacterial Shannon index; SIF, fungal Shannon index; NMDS1B, bacterial the first component of nonmetric multidimensional scaling 1 analysis; NMDS1F, fungal the first component of nonmetric multidimensional scaling 1 analysis; SR10, soil respiration at the 0–10 cm depth; SR50, soil respiration at the 10–50 cm depth; SR100, soil respiration at the 50–100 cm depth. * *p* < 0.05.

**Figure 5:**
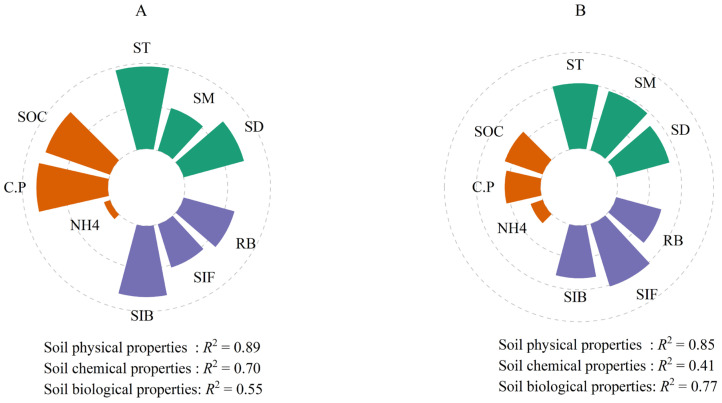
The relative importance of representative factors in regulating soil respiration in growing (**A**) and non-growing (**B**) seasons. SOC, soil organic carbon; C.P, the ratio of soil organic carbon/total phosphorus; NH4, ammonium nitrogen; SD, soil bulk density; ST, soil temperature; SM, soil moisture; RB, root biomass; SIB, bacterial Shannon index; SIF, fungal Shannon index.

Pearson analysis revealed significant correlations between SR and soil chemical properties (NH_4^+^_, C:P, SOC), soil physical properties (SM, ST, SD), and soil microbial characteristics (RB, bacterial Shannon index, fungal Shannon index) (Figure 3 and Figure 4). Hierarchical partitioning showed that soil physical properties contributed most to SR variance (growing: 88.6%; non-growing: 85.3%); followed by soil biological (growing: 79.0%; non-growing: 77.1%) and chemical (growing: 68.4%; non-growing: 41.5%) properties (Figure 5). Additionally, SR showed significant positive correlations with Ascomycota and Thaumarchaeota abundance during growing season, and with Mucoromycota and Acidobacteriota in non-growing season (Figure S1).

### 3.4. Environmental Influences on Soil Respiration at Three Depths

**Figure 6:**
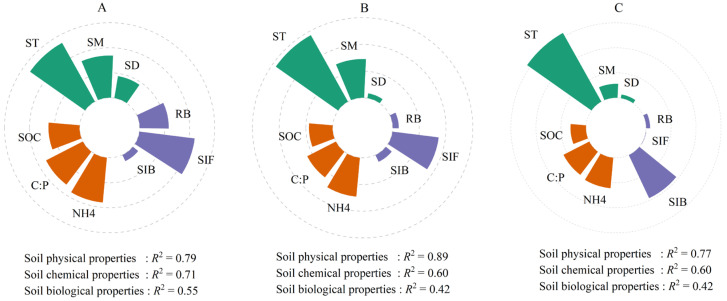
The relative importance of predictive factors in regulating soil respiration at depths of 0–10 (**A**), 10–50 (**B**), and 50–100 cm (**C**). SOC, soil organic carbon; C:P, the ratio of soil organic carbon/total phosphorus; NH4, ammonium nitrogen; SD, soil bulk density; ST, soil temperature; SM, soil moisture; RB, root biomass; SIB, bacterial Shannon index; SIF, fungal Shannon index.

Soil physical properties (ST, SM, SD) explained most variance in SR at the 0–10 (78.9%), 10–50 (89.7%), and 50–100 cm (76.9%), significantly exceeding soil chemical (60.0–70.9%) (SOC, C:P ratio, NH_4^+^_) and biological properties (41.6–54.9%) (RB, bacterial Shannon index, fungal Shannon index) (Figure 6). Depth-specific patterns indicated that the effect of soil chemical properties, physical properties, and biological properties declined with increasing depth. Significant negative correlations (*p* < 0.05) were observed for SR with Mortierellomycota and Gemmatimonadota, contrasting with positive correlations for Ascomycota and Acidobacteriota at the same significance level (Figure S2).

## 4. Discussion

### 4.1. The Soil Respiration During the Nongrowing Season Is Not Negligible

Our findings revealed that soil CO_2_ release during the non-growing season accounted for 32.9% of the annual soil CO_2_ release, a value consistent with the 21.96–34.24% range reported by Chen et al. (2023) [39] for temperate forest ecosystems. Among the controlling factors, soil physical properties (temperature, moisture, bulk density) explained the greatest variation in SR (Figure 5).

Notably, increasing ST stimulates microbial and root activity, accelerates organic matter decomposition, and enhances both microbial and root respiration [40,41]. This conclusion has been widely acknowledged in the ecosystem science community [7,42]. Meanwhile, increasing SM enhanced microbial access to labile C and nutrient diffusion, sustaining higher SR, while limited water availability reduced microbial metabolism and root activity, resulting in lower SR [43]. SD affected SR by altering porosity and gas exchange; higher SD limited root penetration and reduced soil aeration, which in turn restricted SR. In our study, ST and SM decreased significantly, while SD increased from the growing to the non-growing season (Table 1), leading to a marked reduction in SR during the non-growing season.

Soil nutrient availability and elemental stoichiometry have strong influences on SR through regulating microbial decomposition [44]. Notably, abundant nutrients stimulate microbial growth and metabolism, thus accelerating SR [45]. Growing season generally receives higher quantities and more diverse forms of fresh, labile C inputs than non-growing season [46]. The availability of microbial substrates typically decreases during the non-growing season, thereby limiting SR. In this study, SOC and NH_4^+^_ decreased significantly from growing to non-growing season (Table 1). Pearson correlation showed that SR increased significant with NH_4^+^_ and SOC (Figure 3). This phenomenon has been confirmed in various ecosystems [5,47]. Thus, microorganisms exhibited lower metabolic activity due to the lower C availability, resulting in a reduction in SR during non-growing season.

Soil microorganisms, as primary decomposers, drive the SR by mineralizing SOC into CO_2_ through heterotrophic respiration [48]. The growing season, characterized by abundant organic matter and favorable temperature, provides optimal conditions for microbial growth and high SR [49,50]. In contrast, the ability of microbial communities to adapt to environmental stressors is a crucial mechanism enabling the persistence of SR under harsh conditions [51]. For example, Acidobacteriota, a dominant bacterial phylum, exhibits oligotrophic traits and slow growth, conserving energy under lower temperatures by metabolizing recalcitrant organic compounds and maintaining enzyme and membrane functionality [52]. These physiological adaptations of Acidobacteriota have been shown to play a significant role in sustaining microbial activity even when substrate availability is low [53,54]. Similarly, Mucoromycota fungi produce cold-tolerant extracellular enzymes that remain active under lower temperature, enabling the decomposition of complex polymers and providing C to both themselves and co-occurring microbes [55]. In our study, both Mucoromycota and Acidobacteriota showed increased abundance during the non-growing season, with Pearson correlation revealing a significant positive relationship with SR (Figure S1). These findings align with previous studies highlighting the role of cold-adaptive microbial taxa in maintaining microbial activity during winter months [56].

### 4.2. Limited Effect of Soil Depths of Soil Respiration

**Figure 7:**
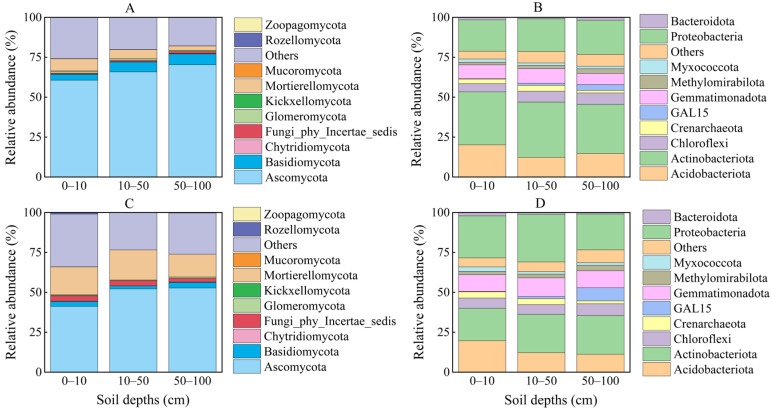
The relative abundances of the fungal (**A**,**C**) and bacterial (**B**,**D**) communities at the phylum level are displayed for the growing (**A**,**B**) and non-growing (**C**,**D**) seasons.

Our findings revealed that SR decreased with increasing depth, a result consistent with previous observations in *Robinia pseudoacacia* plantations in the Loess Plateau hilly region [10]. Although the factors controlling SR in the non-growing season also reflected similar underlying physical, chemical, and biological properties in deep soil, microbial communities played distinct functional roles that directly influenced deep SR (Figure 4 and Figure 6). In our study, the relative abundance of Ascomycota was positively associated with SR (Figure 4) and showed a clear trend of increasing with soil depth (Figure 7). Notably, Ascomycota fungi secrete extracellular enzymes that degrade complex polysaccharides, facilitating nutrient mobilization and enhancing microbial metabolic activity, substrate availability, and nutrient cycling in deep soils [57,58]. As nutrient availability declines with depth, Ascomycota increasingly rely on recalcitrant substrates to support microbial growth [59], a strategy that has been shown to enhance their competitive advantage in low-nutrient environments [60]. This strategy not only explains their higher abundance in deeper soil layers but also highlights their significant role in maintaining microbial activity and supporting continuous CO_2_ production. This finding underscores the importance of microbial functional diversity in soil carbon cycling, particularly in deeper soil layers where nutrient limitations and the availability of substrates may impose constraints on microbial metabolism.

Meanwhile, when ST dropped below 0 °C, no significant correlation was found between ST and SR at a depth of 0–10 cm, with ST explaining only 2% of the variation in SR. In our earlier study, SR in *R. pseudoacacia* stands remained positively correlated with ST even under subzero conditions. This response can be attributed to high nutrient input from persistent litterfall and the contribution of deep roots that remain physiologically active in winter. As a deciduous tree, *R. pseudoacacia* maintains perennial coarse roots and stores substantial non-structural carbohydrates, which can sustain root respiration and fuel microbial metabolism during cold periods [61,62]. Consequently, even slight increases in temperature stimulate microbial activity and organic matter decomposition, resulting in enhanced CO_2_ release [63]. In contrast, *C. korshinskii* stands exhibited no significant correlation between SR and ST below 0 °C. As a shallow-rooted shrub, *C. korshinskii* allocates limited C belowground and its fine roots are largely confined to the upper 10 cm, where frequent freeze–thaw cycles occur. During dormancy, *C. korshinskii* reduces metabolic activity and stores lower levels of non-structural carbohydrates compared with trees, restricting the carbon supply to roots and associated microbes (Table 1). Freeze–thaw damage further impairs root membrane integrity and disrupts rhizodeposition, weakening the coupling between plant carbon inputs and microbial respiration [64]. As a result, despite temperature fluctuations near 0 °C, insufficient root-derived substrates and reduced microbial activity constrain CO_2_ release in *C. korshinskii* stands.

### 4.3. Uncertainty Consideration

The experiment was conducted in the Loess Plateau hilly region, which limits the generalizability of our findings to other ecosystems. Further studies in diverse ecosystems and over larger geographical areas are needed to confirm the applicability of these results to different environmental conditions

Fick’s first law provides a useful framework for estimating SR but has notable limitations, particularly for deep soil SR or seasonal variations. It assumes a uniform soil environment, yet natural soils exhibit significant heterogeneity. As a result, lateral diffusion (horizontal gas movement) can occur in deeper soils, which the law does not account for, potentially underestimating SR in these layers. Additionally, the model’s use of an average diffusion coefficient may overlook microscale variability. Fick’s law also only describes diffusion and does not consider the dynamic production of CO_2_. Measurement errors in boundary conditions can further affect the accuracy of SR estimates.

## 5. Conclusions

Our findings revealed two key insights: (1) cumulative soil CO_2_ fluxes reached 686.9 g CO_2_·m^−2^, with contributions of 510.1, 131.5, and 45.3 g CO_2_·m^−2^·yr^−1^ from the 0–10, 10–50, and 50–100 cm layers, respectively. During the non-growing season, CO_2_ emissions accounted for 33%, 31%, and 32% of the fluxes in 0–10 cm, 10–50 cm, and 50–100 cm depths. This finding highlights the significance of acknowledging CO_2_ release in deep and non-growing seasons. (2) The seasonal and depth-related variations in SR were primarily influenced by the combined effects of soil physical, biological, and chemical properties. However, the significance of these variations differed. These insights suggest that neglecting deep and non-growing season CO_2_ fluxes may cause systematic underestimation of terrestrial C release in current Earth system models. By explicitly accounting for depth-resolved and season-sensitive processes, our study provides a stronger empirical basis for refining terrestrial C feedbacks in climate modeling frameworks.

## Figures and Tables

**Table 1:** Selected soil properties at three depths across the two seasons. Data are presented as mean ± standard error. Capital letters represent seasonal differences; lowercase letters represent differences among soil depths.

Depth (cm)	Growing Season			Non-Growing Season			S	D	S × D
	0–10	10–50	50–100	0–10	10–50	50–100	*p*		
TN (g·kg^−1^)	0.97 ± 0.16 Aa	0.34 ± 0.10 Ab	0.29 ± 0.03 Ab	0.97 ± 0.06 Aa	0.34 ± 0.07 Ab	0.30 ± 0.05 Ab	0.24	<0.01	0.87
NH_4^+^_ (g·kg^−1^)	0.10 ± 0.06 Aa	0.09 ± 0.05 Aa	0.08 ± 0.10 Aa	0.07 ± 0.03 Ba	0.054 ± 0.11 Ba	0.05 ± 0.05 Ba	<0.01	0.43	0.38
TP (g·kg^−1^)	0.64 ± 0.00 Aa	0.62 ± 0.00 Aa	0.61 ± 0.00 Aa	0.56 ± 0.01 Ba	0.55 ± 0.02 Ba	0.54 ± 0.00 Ba	<0.01	0.88	0.32
pH	8.45 ± 0.14 Aa	8.49 ± 0.16 Aa	8.6 ± 0.08 Aa	8.43 ± 0.18 Aa	8.57 ± 0.06 Aa	8.72 ± 0.22 Aa	0.41	0.13	0.73
SOC (g·kg^−1^)	5.64 ± 0.27 Aa	3.88 ± 0.97 Ab	2.92 ± 0.17 Ab	4.68 ± 0.25 Aa	3.59 ± 0.84 Ab	2.91 ± 0.42 Ab	0.31	<0.05	0.78
N:P	1.52 ± 0.25 Aa	0.55 ± 0.03 Ab	0.48 ± 0.17 Ab	1.73 ± 0.11 Aa	0.62 ± 0.13 Ab	0.56 ± 0.09 Ab	0.7	<0.01	<0.01
NO_3^−^_ (g·kg^−1^)	0.58 ± 0.11 Aa	0.47 ± 0.63 Aa	0.27 ± 0.31 Aa	0.53 ± 0.67 Aa	0.30 ± 0.25 Aa	0.23 ± 0.20 Aa	0.62	0.85	0.34
C:P	8.81 ± 0.71 Aa	6.25 ± 1.63 Ab	4.79 ± 0.48 Ab	8.38 ± 0.36 Ba	6.53 ± 1.37 Ab	5.39 ± 0.67 Ab	0.21	<0.01	<0.01
C:N	5.81 ± 1.17 Aa	11.4 ± 1.58 Ab	10.1 ± 0.29 Ab	4.81 ± 0.53 Aa	10.6 ± 4.64 Ab	9.7 ± 0.70 Ab	0.448	<0.01	0.08
SD (g·cm^−3^)	1.35 ± 0.03 Aa	1.45 ± 0.03 Ab	1.58 ± 0.01 Ac	1.37 ± 0.03 Aa	1.47 ± 0.03 Ab	1.60 ± 0.01 Ac	0.27	<0.05	0.51
ST (°C)	15.9 ± 0.33 Aa	14.9 ± 0.40 Ab	13.6 ± 0.33 Ac	−0.3 ± 0.21 Ba	0.93 ± 0.15 Bb	3.12 ± 0.20 Bc	<0.01	<0.01	<0.01
SM (cm^3^·cm^−3^)	0.10 ± 0.00 Aa	0.12 ± 0.02 Aa	0.12 ± 0.01 Aa	0.05 ± 0.00 Ba	0.09 ± 0.01 Ba	0.12 ± 0.01 Ba	<0.01	<0.01	<0.01
RB	943.15 ± 59.9 Aa	563.12 ± 69.0 Ab	409.42 ± 77.1 Ac	754.91 ± 87.5 Ba	532.85 ± 6.50 Bb	370.73 ± 61.1 Bc	<0.01	<0.05	<0.05
MBC (g·kg^−1^)	14.46 ± 0.894 Aa	9.53 ± 6.38 Ab	8.23 ± 5.97 Ac	13.09 ± 1.58 Aa	8.67 ± 4.26 Ab	6.46 ± 0.43 Ac	0.43	<0.05	0.91
SIF	5.98 ± 0.02 Aa	5.38 ± 0.02 Ab	4.95 ± 0.01 Ac	7.14 ± 0.02 Ba	5.11 ± 0.01 Bb	4.95 ± 0.00 Bc	<0.01	<0.01	0.12
SIB	10.4 ± 0.11 Aa	9.75 ± 0.10 Ab	9.54 ± 0.11 Ac	10.2 ± 0.10 Aa	9.73 ± 0.08 Ab	9.04 ± 0.07 Ac	0.81	<0.05	0.63
NMDS1B	−0.10 ± 0.02 Aa	−0.05 ± 0.14 Ab	0.01 ± 0.11 Ab	−0.10 ± 0.07 Aa	0.00 ± 0.07 Ab	0.06 ± 0.018 Ab	0.31	<0.05	0.07
NMDS1F	−0.49 ± 0.17 Aa	−0.25 ± 0.14 Ab	−0.12 ± 0.18 Ac	−0.20 ± 0.03 Ba	−0.13 ± 0.27 Bb	0.66 ± 0.082 Bc	<0.05	<0.01	<0.05

Note: TN, total nitrogen; NH_4^+^_, ammonium nitrogen; TP, soil total phosphorus; pH, potential of Hydrogen; SOC, soil organic carbon; N:P, the ratio of total nitrogen/total phosphorus; NO_3^−^_, nitrate nitrogen; C:P, the ratio of soil organic carbon/total phosphorus; C:N, the ratio of soil organic carbon/total nitrogen; SD, soil bulk density; ST, soil temperature; SM, soil moisture; RB, root biomass; MBC, microbial biomass carbon; SIB, bacterial Shannon index; SIF, fungal Shannon index; NMDS1B, bacterial the first component of nonmetric multidimensional scaling (NMDS) 1 analysis; NMDS1F, fungal the first component of nonmetric multidimensional scaling (NMDS) 1 analysis; S, seasons; D, soil depths; S × D, interactions between seasons and soil depths; *p*, statistical significance.

## Data Availability

The data from this study are available upon request to the corresponding author. The data are not publicly available due to privacy restrictions.

## Supplementary Materials

The following supporting information can be downloaded at https://www.mdpi.com/article/10.3390/plants14193038/s1, Figure S1: Correlations of soil respiration with the relative abundances of major fungal and bacterial phyla during the growing and non-growing seasons. GSR, soil respiration during the growing season; NGSR, soil respiration during the non-growing season. * *p* < 0.05; ** *p* < 0.01. Figure S2 Correlations of soil respiration with the relative abundances of major fungal and bacterial phyla in 0–10 cm, 10–50 cm, and 50–100 cm soil depths. SR10, soil respiration in 0–10 cm soil depth; SR50, soil respiration in 10–50 cm soil depth; SR100, soil respiration in 50–100 cm soil depth. * *p* < 0.05; ** *p* < 0.01. Table S1: Relationship between soil respiration and soil temperature at three depths between two seasons. Table S2: Relationship between soil respiration and soil moisture at three depths between two seasons. Table S3: Soil respiration as a function of soil moisture and soil temperature at three depths between two periods.

## References

[1] Zhang J., Li P.K., Li L., Zhao M.N., Yan P.S., Liu Y., Li W., Ding S.Y., Zhao Q.H. (2025). Soil respiration and carbon sequestration response to short-term fertilization in wheat-maize cropping system in the North China Plain. Soil Tillage Res..

[2] Liu Z.H., Huang F.Y., Wang B.F., Li Z.Y., Zhao C.X., Ding R.X., Yang B.P., Zhang P., Jia Z.K. (2023). Soil respiration in response to biotic and abiotic factors under different mulching measures on rain-fed farmland. Soil Tillage Res..

[3] Yang Z.H., Luo X.R., Shi Y.H., Zhou T., Luo K., Lai Y.S., Yu P., Liu L., Olchev A., Bond-Lamberty B. (2023). Controls and variability of soil respiration temperature sensitivity across China. Sci. Total Environ..

[4] Chen H.L., Gou M.M., Hu J.W., Lei L., Zhu S.F., Hu R.Y., Zhao H.P., Xiao W.F., Liu C.F. (2024). Seasonal variations in soil enzyme activity and nutrient limitations of differently aged Pinus massoniana plantation. Microorganisms.

[5] Han Y.J., Wang G.S., Zhou S.H., Li W.Y., Xiong L.H. (2024). Day-night discrepancy in soil respiration varies with seasons in a temperate forest. Funct. Ecol..

[6] Laza H.E., Acosta-Martinez V., Cano A., Baker J., Mahan J., Gitz D., Emendack Y., Slaughter L., Lascano R., Tissue D. (2023). Elevated [CO_2_] enhances soil respiration and AMF abundance in a semiarid peanut agroecosystem. Agric. Ecosyst. Environ..

[7] Abdalla K., Schierling L., Sun Y., Schuchardt M.A., Jentsch A., Deola T., Wolff P., Kiese R., Lehndorff E., Pausch M. (2024). Temperature sensitivity of soil respiration declines with climate warming in subalpine and alpine grassland soils. Biogeochemistry.

[8] Cordeiro A.L., Cusack D.F., Dietterich L.H., Hockaday W.C., Mcfarlane K.J., Sivapalan V., Hedgpeth A., Neupane A., Colburn L., Konwent W. (2024). Root characteristics vary with depth across four lowland seasonal tropical forests. Ecosystems.

[9] Zhang Q.F., Qin W.K., Feng J.G., Li X.J., Zhang Z.H., He J.S., Schimel J.P., Zhu B. (2023). Whole-soil-profile warming does not change microbial carbon use efficiency in surface and deep soils. Proc. Natl. Acad. Sci. USA.

[10] Sun Y.R., Lu S.B., Chen Y.M. (2025). Variations and controlling factors of soil CO_2_ release at daytime and nighttime scales in the loess hilly regions of China. Geoderma.

[11] Muratore T.J., Knorr M.A., Simpson M.J., Stephens R.B., Phillips R.P., Frey S.D. (2024). Response of Root Respiration to Warming and Nitrogen Addition Depends on Tree Species. Glob. Change Biol..

[12] Guan C., Zhao C.M., Dacal M., Gozalo B., Ochoa V., Asensio S., Corrochano-Monsalve M., Chen N., Biancari L., Maestre F.T. (2025). Biocrusts alter the effects of long-term warming on soil respiration in a dryland ecosystem. Geoderma.

[13] Liu S., Li X., Fu Y.J., Li P., Qiao J., Li H., Wu L.C., Wang B.P., Lu S. (2024). Exploring the effects of different fertilizer application durations on the functional microbial profiles of soil carbon and nitrogen cycling by using metagenomics in Paulownia plantations in a subtropical zone. Eur. J. For. Res..

[14] Luboš S., Jindřich N., Radomír U. (2025). Changes in the concentration of CO_2_ in forest soils resulting from the traffic of logging machines. J. For. Sci..

[15] Podzikowski L.Y., Billings S.A., Bever J.D. (2025). Plant functional diversity shapes soil respiration response to soil moisture availability. Ecosystems.

[16] Zveushe O.K., Sajid S., Dong F.Q., Han Y., Zeng F., Geng Y.H., Shen S.R., Xiang Y.L., Kang Q.L., Zhang Y.Z. (2025). Different sex combinations of Populus cathayana affect soil respiration and tea litter decomposition by influencing plant growth and soil functional microbial diversity. Plant Soil.

[17] Wen S.H., Delgado-Baquerizo M., Saez-Sandino T., Chen J.Y., Feng J., Huang Q.Y., Guirado E., Rillig M.C., Liu Y.R. (2025). Negative impacts of global change stressors permeate into deep soils. Ecol. Lett..

[18] Yan Y.X., Shi J., Fan Z.M., Peng Y.M., Wang X. (2025). Changes in long-term land use alter deep soil microbial necromass and organic carbon stabilization. J. Environ. Manag..

[19] Zuo Y.L., He C., Zhang D.D., Zhao L.L., He X.L., Sun X. (2023). Soil variables driven by host plant and growth season affect soil microbial composition and metabolism in extremely arid desert ecosystems. Microbiol. Res..

[20] Yang Y., Gunina A., Chen J., Wang B.R., Cheng H., Wang Y.Q., Liang C., An S.S., Chang S.X., Delgado-Baquerizo M. (2025). Unfolding the Potential of Soil Microbial Community Diversity for Accumulation of Necromass Carbon at Large Scale. Glob. Change Biol..

[21] Jian C.X., Luo Y., Zhou J.J., Xu B.C. (2024). Variation Patterns and Affecting Factors of Plant Alpha Diversity, Beta Diversity and Its Components in Restoration Grasslands on Loess Plateau. Land Degrad. Dev..

[22] Zhou J.J., Chen Z.F., Jian C.X., Luo Y., Niu F.R., Palta J.A., Xu B.C. (2024). Autotrophic Respiration Is More Sensitive to Nitrogen and Phosphorus Supply Than Heterotrophic Respiration in Semiarid Grassland. J. Geophys. Res. Biogeosci..

[23] Luo Y., Chen Y., Jian C.X., Zhou J.J., Mou Y.K., Jin Y., Wang S.Y., Xu B.C. (2025). Effects of surface vegetation and litter on rainfall redistribution during the rainy season in semiarid grasslands. J. Hydrol..

[24] Sun Y.R., Liu C., Zhao M., Liu L., Liang S.Q., Wang Y.J., Chen Y.M. (2023). Influence of extreme rainfall events on soil carbon release in the Loess Hilly Region, China. Catena.

[25] Xu H., Wang Z.J., Li Y., He J.L., Wu X.D. (2020). Dynamic growth models for Caragana korshinskii shrub biomass in China. J. Environ. Manag..

[26] Chen Z.F., Xiong P.F., Zhou J.J., Lai S.B., Jian C.X., Xu W.Z., Xu B.C. (2021). Effects of plant diversity on semiarid grassland stability depend on functional group composition and dynamics under N and P addition. Sci. Total Environ..

[27] FAO (2020). Global Forest Resources Assessment.

[28] Yang X., Wang R., Yang M.D., Liu Q.F., Zhang W.J., Guo S.L. (2025). Differential responses of soil CO_2_ dynamics along soil depth to rainfall patterns in the Chinese Loess Plateau. Agric. Ecosyst. Environ..

[29] Contosta A.R., Burakowski E.A., Varner R.K., Frey S.D. (2016). Winter soil respiration in a humid temperate forest: The roles of moisture, temperature, and snowpack. J. Geophys. Res. Biogeosci..

[30] Carter M.R., Gregorich E.G. (2007). Soil Sampling and Methods of Analysis.

[31] Zhao M., Sun Y.R., Liu S.H., Li Y.C., Chen Y.M. (2024). Effects of stand density on the structure of soil microbial functional groups in Robinia pseudoacacia plantations in the hilly and gully region of the Loess Plateau, China. Sci. Total Environ..

[32] Maier M., Schack-Kirchner H. (2014). Using the gradient method to determine soil gas flux: A review. Agric. For. Meteorol..

[33] Rühlmann J., Körschens M., Graefe J. (2006). A new approach to calculate the particle density of soils considering properties of the soil organic matter and the mineral matrix. Geoderma.

[34] Zhang Q., Phillips R.P., Manzoni S., Scott R.L., Oishi A.C., Finzi A.C., Daly E., Vargas R., Novick K.A. (2018). Changes in photosynthesis and soil moisture drive the seasonal soil respiration-temperature hysteresis relationship. Agric. For. Meteorol..

[35] Xu M., Qi Y. (2001). Soil-surface CO_2_ efflux and its spatial and temporal variations in a young ponderosa pine plantation in northern California. Glob. Change Biol..

[36] Shi W.Y., Du S., Morina J.C., Guan J.H., Wang K.B., Ma M.G., Yamanaka N., Tateno R. (2018). Physical and biogeochemical controls on soil respiration along a topographical gradient in a semiarid forest. Agric. For. Meteorol..

[37] Davidson E.A., Samanta S., Caramori S.S., Savage K. (2012). The dual Arrhenius and Michaelis-Menten kinetics model for decomposition of soil organic matter at hourly to seasonal time scales. Glob. Change Biol..

[38] Lai J.S., Zou Y., Zhang J.L., Peres-Neto P.R. (2022). Generalizing hierarchical and variation partitioning in multiple regression and canonical analyses using the rdacca.hp R package. Methods Ecol. Evol..

[39] Chen L.F., Yang S.P., He Z.B., Zhao W.Z., Kong J.Q., Feng X.Y., Li X.G. (2023). Divergent seasonal patterns and drivers of soil respiration in alpine forests of northwestern China. Agric. For. Meteorol..

[40] Brown R., Markewitz D. (2018). Soil heterotrophic respiration: Measuring and modeling seasonal variation and silvicultural impacts. For. Ecol. Manag..

[41] Liu Y.C., Wang H., Schindlbacher A., Liu S.R., Yang Y.J., Tian H.M., Chen L., Ming A.G., Wang J., Li J.C. (2025). Soil respiration related to the molecular composition of soil organic matter in subtropical and temperate forests under soil warming. Soil Biol. Biochem..

[42] Dacal M., Delgado-Baquerizo M., Barquero J., Berhe A.A., Gallardo A., Maestre F.T., García-Palacios P. (2022). Temperature increases soil respiration across ecosystem types and soil development, but soil properties determine the magnitude of this effect. Ecosystems.

[43] Lin J.J., Chen B.B., Dong H.X., Zhang W.L., Kumar A., Hui D.F., Zhang C.G., Shan S.D., Zhu B. (2025). Effects of soil moisture fluctuation and microplastics types on soil organic matter decomposition and carbon dynamics. Soil Biol. Biochem..

[44] Soong J.L., Marañon-Jimenez S., Cotrufo M.F., Boeckx P., Bodé S., Guenet B., Peñuelas J., Richter A., Stahl C., Verbruggen E. (2018). Soil microbial CNP and respiration responses to organic matter and nutrient additions: Evidence from a tropical soil incubation. Soil Biol. Biochem..

[45] Nguyen T.T., Marschner P. (2016). Soil respiration, microbial biomass and nutrient availability in soil after repeated addition of low and high C/N plant residues. Biol. Fertil. Soils.

[46] Xue X.P., Ge X.G., Lei L., Zhou B.Z., Li M.H. (2024). Effects of phosphorus resorption on bioactive phosphorus of different-aged Pinus massoniana plantations. For. Ecosyst..

[47] Hursh A., Ballantyne A., Cooper L., Maneta M., Kimball J., Watts J. (2017). The sensitivity of soil respiration to soil temperature, moisture, and carbon supply at the global scale. Glob. Change Biol..

[48] Duan P.P., Fu R.T., Nottingham A.T., Domeignoz-Horta L.A., Yang X.Y., Du H., Wang K.L., Li D.J. (2023). Tree species diversity increases soil microbial carbon use efficiency in a subtropical forest. Glob. Change Biol..

[49] Wang H.H., Huang W.D., He Y.Z., Zhu Y.Z. (2023). Effects of warming and precipitation reduction on soil respiration in Horqin sandy grassland, northern China. Catena.

[50] Guan C., Song X.Y., Zhou S.Y., Jiang Y.F., Qiao L.J., Ma X.J., Chen N., Zhao C.M. (2025). Divergent responses of soil respiration to biocrusts during the nongrowing and growing seasons in a dryland shrubland ecosystem. Appl. Soil Ecol..

[51] Li G.Y., Mu J.P., Liu Y.Z., Smith N.G., Sun S.C. (2017). Effect of microtopography on soil respiration in an alpine meadow of the Qinghai-Tibetan plateau. Plant Soil.

[52] Zeng Q.Q., Lyu X. (2025). Identification of a novel cellobiose 2-epimerase from Acidobacteriota bacterium and its application for in-situ milk catalysis. Front. Microbiol..

[53] Goncalves O.S., Fernandes A.S., Tupy S.M., Ferreira T.G., Almeida L.N., Creevey C.J., Santana M.F. (2024). Insights into plant interactions and the biogeochemical role of the globally widespread Acidobacteriota phylum. Soil Biol. Biochem..

[54] Liu M.H., Feng F.J., Cai T.J., Tang S.J. (2020). Soil Microbial Community Response Differently to the Frequency and Strength of Freeze-Thaw Events in a Larix gmelinii Forest in the Daxing’an Mountains, China. Front. Microbiol..

[55] Klink S., Keller A.B., Wild A.J., Baumert V.L., Gube M., Lehndorff E., Meyer N., Mueller C.W., Phillips R.P., Pausch J. (2022). Stable isotopes reveal that fungal residues contribute more to mineral-associated organic matter pools than plant residues. Soil Biol. Biochem..

[56] Christiansen C.T., Engel K., Hall M., Neufeld J.D., Walker V.K., Grogan P. (2024). Arctic tundra soil depth, more than seasonality, determines active layer bacterial community variation down to the permafrost transition. Soil Biol. Biochem..

[57] Wu L.F., Zhou L.H., Zou B.Z., Wang S.R., Zheng Y., Huang Z.Q., He J.Z. (2022). Soil Fungal Diversity and Functionality Changes Associated with Multispecies Restoration of Pinus massoniana Plantation in Subtropical China. Forests.

[58] Moulin V., Henneron L., Ribémont R., Colin Y., Buquet S., Vincenot L., Aubert M. (2025). Standing at the crossroads: Path analysis highlights potential levers to preserve fungal richness when shifting tree species for forest adaptation. For. Ecol. Manag..

[59] He X., Yang T., Rinne-Garmston K.T., Ji X.Y., Xu Q., Xu Z.Y., Zheng Y.X., Zhao Z.Q., Zhao G.P., Hu Z.H. (2025). Wood-inhabiting fungal community characteristics responses to nutrient additions vary among tree taxonomic groups. J. Soil Sediments.

[60] Zhou J., Li Y.L., Lou J.W., Wang Y.K., Kan Z.R., Neugschwandtner R.W., Li F.M., Liu J., Dong K., Xue Y.G. (2024). Fungal Saprotrophic Promotion and Plant Pathogenic Suppression under Ditch-Buried Straw Return with Appropriate Burial Amount and Depth. Plant.

[61] Nola P., Bracco F., Assini S., von Arx G., Castagneri D. (2020). Xylem anatomy of *Robinia pseudoacacia* L. and *Quercus robur* L. is differently affected by climate in a temperate alluvial forest. Ann. For. Sci..

[62] Dong C.G., Qiao Y.N., Cao Y., Chen Y.M., Wu X., Xue W.Y. (2021). Seasonal variations in carbon, nitrogen, and phosphorus stoichiometry of a *Robinia pseudoacacia* plantation on the Loess Hilly Region, China. Forests.

[63] Sun Y.R., Zhao M., Liu L., Liu S.H., Dong C.G., Chen Y.M. (2024). Plant carbon removal affects monthly temperature sensitivity of soil respiration during growing season in three typical plantations in the Loess Hilly Region, China. Catena.

[64] Pastore M.A., Classen A.T., English M.E., Frey S.D., Knorr M.A., Rand K., Adair E.C. (2023). Soil microbial legacies influence freeze-thaw responses of soil. Funct. Ecol..

