# Supplementary material for: Depth and Seasonality of Soil Respiration in Caragana korshinskii Plantation on the Loess Plateau
Source: Plants (Basel). 2025 Oct 1;14(19):3038. doi: 10.3390/plants14193038 (PMC12525916; doi:10.3390/plants14193038)
Supplement: Supplementary file 1 [file plants-14-03038-s001.zip › plants-3865601-supplementary.pdf]

Supplementary File

Materials and methods

Soil sampling and analysis

DNA was extracted from soil samples using the cetyl trimethyl ammonium bromide (CTAB) method to ensure efficient recovery of high-quality microbial DNA. The concentration and purity of extracted DNA were evaluated on a 1% agarose gel. For microbial community profiling, the V4 region of the bacterial 16S rRNA gene was amplified using primers 515F and 806R, chosen for their broad coverage of bacterial taxa, while the fungal ITS1 region was targeted using primers ITS5-1737F and ITS2-2043R to capture fungal diversity. PCR products were visualized on a 2% agarose gel with SYBR Green staining to confirm successful amplification, pooled in equimolar ratios, and purified using the QIAquick Gel Extraction Kit (Qiagen, Germany). Sequencing libraries were prepared with the TruSeq® DNA PCR-Free Sample Preparation Kit (Illumina, USA), incorporating unique index codes for each sample. Library quality was assessed with a Qubit@2.0 Fluorometer and Agilent Bioanalyzer 2100 to ensure compliance with sequencing standards. High-throughput sequencing was conducted on an Illumina NovaSeq platform (Novogene, Beijing, China), generating paired-end reads for comprehensive microbial community analysis. Subsequent data processing included quality filtering, OTU clustering at 97% similarity thresholds, and taxonomic annotation against the SILVA (for bacteria) and UNITE (for fungi) reference databases, providing robust assessments of microbial diversity and composition. Bacterial and fungal α (richness) diversity were calculated based on 97% operational taxonomic units (OTUs) similarity of obtained bacterial and fungal sequences. Microbial composition was represented by the first component of nonmetric multidimensional scaling (NMDS) analysis

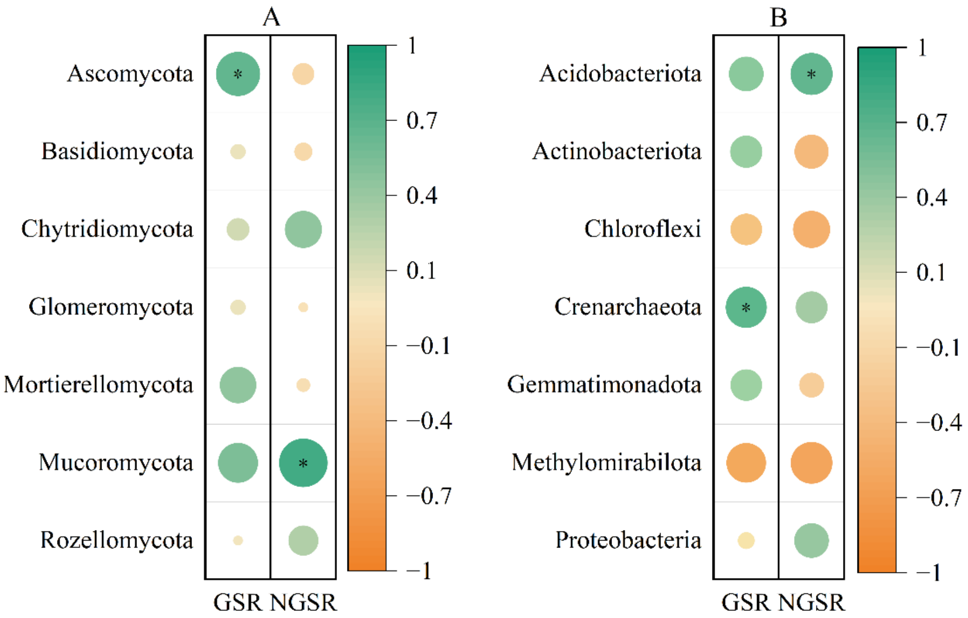

Figure. S1 Correlations of soil respiration with the relative abundances of major fungal and bacterial phyla during the growing and non-growing seasons. GSR, soil respiration during the growing season; NGSR, soil respiration during the non-growing season. *$p < 0.05$; **$p < 0.01$.

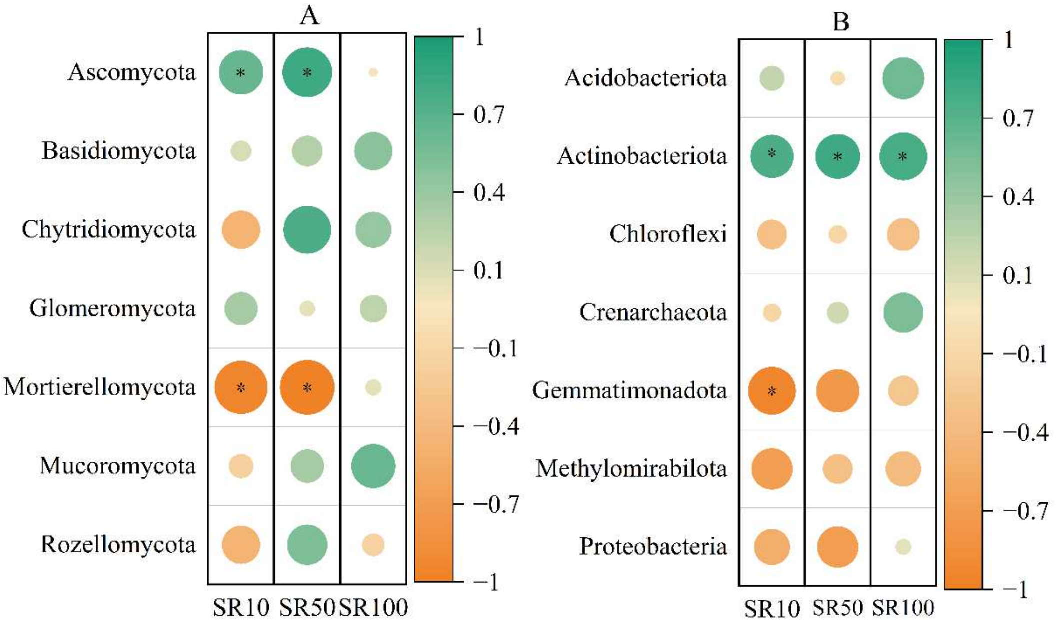

Figure. S2 Correlations of soil respiration with the relative abundances of major fungal and bacterial phyla in 0–10 cm, 10–50 cm, and 50–100 cm soil depths. SR10, soil respiration in 0–10 cm soil depth; SR50, soil respiration in 10–50 cm soil depth; SR100, soil respiration in 50–100 cm soil depth. $*p < 0.05$; $**p < 0.01$.

Table S1: Relationship between soil respiration and soil temperature at three depths between two seasons.

Soil depth	Seasons	a	b	R^2	*p*	ST
0–10	Growing	1.37	0.06	0.45	**<0.01**	>0°C
	Non-growing	1.73	0.06	0.38	**<0.01**	>0°C
		2.03	–0.02	0.02	0.52	<0°C
10–50	Growing	0.91	0.09	0.54	**<0.01**	>0°C
	Non-growing	1.09	0.06	0.38	**<0.01**	>0°C
		1.84	0.36	0.66	**<0.01**	<0°C
50–100	Growing	0.42	0.14	0.77	**<0.01**	>0°C
	Non-growing	0.22	0.22	0.77	**<0.01**	>0°C

Note: ST, soil temperature; *p*, statistical significance.

Table S2: Relationship between soil respiration and soil moisture at three depths between two seasons.

Soil depth	Seasons	a	b	c	R^2	*p*
0–10	Growing	–151.94	30.00	2.57	0.02	0.54
	Non-growing	–371.11	49.61	0.52	0.12	**<0.05**
10–50	Growing	–305.83	48.61	2.26	0.14	**<0.05**
	Non-growing	–673.46	114.75	–2.74	0.56	**<0.01**
50–100	Growing	–42.52	–11.64	5.30	0.36	**<0.01**
	Non-growing	4483.4	–1087.8	66.30	0.38	**<0.01**

Table S3: Soil respiration as a function of soil moisture and soil temperature at three depths between two periods.

Soil depth	Seasons	a	b	d	e	c	R^2	*p*	ST
0–10	Growing	0.564	0.082	14.629	10.542	0.662	0.687	**<0.01**	–
	Non-growing	1.604	0.007	400.121	-84.938	5.505	0.105	0.08	< 0°C
		2.768	-0.061	-587.307	66.215	-0.990	0.654	**<0.01**	>0 °C
10–50	Growing	0.301	0.116	0.306	23.168	-1.1286	0.831	**<0.01**	–
	Non-growing	3.61	0.446	222.458	-54.299	3.315	0.700	**<0.01**	>0 °C
		0.252	0.045	-263.79	61.435	-0.999	0.899	**<0.01**	<0 °C
50–100	Growing	0.203	0.113	-5.983	3.160	0.161	0.898	**<0.01**	–
	Non-growing	4.912	0.086	25.309	-6.594	-6.594	0.793	**<0.01**	–

Note: ST, soil temperature; *p*, statistical significance.
